# Brain Rhythms of Pain

**DOI:** 10.1016/j.tics.2016.12.001

**Published:** 2017-02

**Authors:** Markus Ploner, Christian Sorg, Joachim Gross

**Affiliations:** 1 Department of Neurology and TUMNeuroimaging Center, Technische Universität München, Munich, Germany; 2 Departments of Neuroradiology and Psychiatry and TUMNeuroimaging Center, Technische Universität München, Munich, Germany; 3 Institute of Neuroscience and Psychology, University of Glasgow, Glasgow, UK

**Keywords:** pain, brain, oscillations, information flow, predictive coding

## Abstract

Pain is an integrative phenomenon that results from dynamic interactions between sensory and contextual (i.e., cognitive, emotional, and motivational) processes. In the brain the experience of pain is associated with neuronal oscillations and synchrony at different frequencies. However, an overarching framework for the significance of oscillations for pain remains lacking. Recent concepts relate oscillations at different frequencies to the routing of information flow in the brain and the signaling of predictions and prediction errors. The application of these concepts to pain promises insights into how flexible routing of information flow coordinates diverse processes that merge into the experience of pain. Such insights might have implications for the understanding and treatment of chronic pain.

## Trends

Pain is a vital phenomenon that depends on the dynamic integration of sensory and contextual processes. In chronic pain the adaptive integration of sensory and contextual processes is severely disturbed.

Neuronal oscillations and synchrony at different frequencies provide evidence on information flow across brain areas. The flexible relationship between oscillations at different frequencies and pain indicates flexible routing of information flow in the cerebral processing of pain.

The systematic assessment of oscillations and synchrony in the processing of pain provides insights into how sensory and contextual processes are flexibly integrated into a coherent percept and into abnormalities of these processes in chronic pain. Predictive coding frameworks might help us understand these integration processes.

## How Can the Study of Brain Rhythms Advance Our Understanding of Pain?

Pain results from dynamic interactions between sensory and contextual (i.e., cognitive, emotional, and motivational) processes [1]. Pain is thus essentially an integrative phenomenon. In recent years it has been shown that oscillations and synchrony serve integrative functions by flexibly routing information flow in the brain 2, 3, 4, 5, 6. Thus, understanding the role of oscillations in the processing of pain promises insights into how functionally diverse processes dynamically merge into the experience of pain in health and disease.

Here we review recent evidence on the role of neuronal oscillations and synchrony in the processing of pain. We begin with a brief discussion of the peculiarities of pain and its processing in the brain. We then summarize recent insights into the significance of neuronal oscillations and synchrony for the routing of information flow in the brain. On this basis we review evidence on the role of oscillations in the processing of pain. We specifically discuss how oscillations and synchrony serve the flexible routing of information flow in the integration of sensory and contextual factors into a coherent percept. Moreover, we review and discuss the role of these processes in pathological abnormalities of the pain experience in chronic pain. Finally, we consider perspectives and future directions for the study of the role of neuronal oscillations in the cerebral processing of pain.

## Pain

Pain is an unpleasant sensory and emotional experience that signals threat and promotes behavior to protect the individual. Commonly, the underlying process is that a noxious stimulus induces physiological processes, referred to as **nociception** (see Glossary), that translate into pain [1]. This translation process is influenced by a broad variety of contextual factors. We routinely make use of this influence; for example, when comforting an injured child or when harnessing placebo effects for pain therapy. Pain thus results from the integration of **nociceptive** and contextual information mediated by feedforward and feedback processes in the human brain [7]. This integration process is not static but has to be dynamically adjusted to the continuously changing demands of everyday life. For example, the same noxious input can yield no pain when a competing goal has to be achieved (e.g., during a long-distance run) but under other contextual conditions can result in strong pain (e.g., when a severe disease is feared). Thus, the dynamic integration of sensory and contextual processes plays a preeminent role in pain that probably exceeds its role in other modalities.

In chronic pain states, pain often persists without objective threat to the body. Chronic pain is a disease in its own right that affects about a fifth of the adult population in the Western world 8, 9, imposes an enormous economic burden on society 10, 11, and causes severe suffering to individuals. In chronic pain the relationship between nociception and pain is often weak or lost [12] indicating abnormal integration of nociceptive and contextual information. In particular, there is a close and mutual relationship between contextual factors and chronic pain [13]. For example, certain psychological factors such as passive coping strategies predispose to the development of chronic pain and, conversely, chronic pain yields severe cognitive, affective, and functional deficits [13]. Thus, the adaptive integration of nociceptive and contextual processes is severely disturbed in chronic pain.

## Pain and the Brain

Pain is associated with the activation of an extended network of brain areas including the somatosensory, insular, cingulate, and prefrontal cortices, the thalamus, subcortical areas, and the brainstem [14]. These areas do not constitute a dedicated pain system but belong to different functional systems of the brain that are transiently orchestrated in the processing of pain. None of these areas exclusively processes or singularly determines the experience of pain [15] (see 16, 17, 18, 19 for an ongoing discussion of this topic). It is thus likely that the integration of neuronal activity across brain areas eventually determines pain. Structural connections represent the anatomical basis for this integration process. However, to continuously adjust pain to the momentary behavioral demands, the integration process has to be highly flexible. This flexibility requires dynamic changes of neuronal integration at timescales that can be provided not by changes of structural connections but rather by dynamic changes of functional connections. Such dynamic changes of functional connections in the processing of pain have recently been conceptualized as the dynamic pain connectome [20]. This concept does not conceive the cerebral processing of pain as a static phenomenon but emphasizes that the dynamics of functional connections flexibly determine the experience of pain.

Pain is associated not only with a spatially extended network of dynamically recruited brain areas but also with complex temporal–spectral patterns of brain activity. In particular, pain-related neuronal oscillations at frequencies ranging from infraslow fluctuations below 0.1 Hz (Box 1) via theta (4–7 Hz), alpha (8–13 Hz), and beta (14–29 Hz) to gamma (30–100 Hz) oscillations 21, 22, 23, 24, 25, 26, 27, 28, 29, 30, 31 have been observed. These oscillations have been recorded during different contextual conditions and at different timescales. However, an overarching framework for the significance of these oscillations for pain remains lacking.

The close relationship between chronic pain and psychological factors [13] and the substantial comorbidity of chronic pain and mental disorders [32] indicates that brain dysfunction plays a central role in the development and maintenance of chronic pain. Recent neurobiological investigations corroborate the crucial role of the brain in chronic pain by showing substantial structural and metabolic changes of the brain in chronic pain 12, 33. Moreover, neurophysiological and functional imaging studies found abnormalities of the frequency spectrum of brain activity ranging from infraslow fluctuations (Box 1) to gamma oscillations in patients with chronic pain 26, 27, 34, 35. Most recent evidence indicates that some of these changes are causally involved in the development and maintenance of chronic pain 36, 37.

## Neuronal Oscillations and Synchrony

Brain rhythms or brain oscillations refer to rhythmic fluctuations of neural mass signals recorded by local field potentials (LFPs), electroencephalography (EEG), or magnetoencephalography (MEG) [38]. Brain oscillations are most prominent at frequencies between 1 and 100 Hz [39]. They originate from the dynamic interplay of excitation and inhibition of neuronal populations leading to periodic synchronization of action potentials. In addition, infraslow fluctuations of brain activity are observed at frequencies below 0.1 Hz by functional magnetic resonance imaging (fMRI) (Box 1). At any frequency the synchronization of brain activity can occur both within and between brain areas 40, 41. Brain oscillations have been observed in association with a broad variety of perceptual, cognitive, and behavioral functions. The interpretation of their functional significance therefore varies substantially between tasks and backgrounds. It is only recently that these different interpretations have been complemented by a unified physiological framework indicating that brain oscillations are mechanistically involved in the dynamic routing of information flow 2, 3, 4, 5, 6.

This framework is based on a convergence of anatomical and functional findings in animals and humans. First, in the visual system anatomical connections have been differentiated into feedforward (bottom-up) and feedback (top-down) connections 42, 43. This anatomical differentiation is apparent in distinct distributions of both types of connections across the various layers of the cortex. Feedforward projections typically start in **supragranular layers** and terminate in layer IV. Feedback projections predominantly start in **infragranular layers** and terminate in layers other than layer IV. Second, the non-homogeneous distribution of feedforward and feedback connections is complemented by a non-homogeneous distribution of brain oscillations across cortical layers. Several studies demonstrate that oscillations at alpha and beta frequencies (8–29 Hz) are stronger in infragranular layers than in supragranular layers. By contrast, oscillations in the gamma frequency band (∼30–100 Hz) are stronger in supra- than in infragranular layers of the cortex 44, 45, 46, 47. In light of the aforementioned laminar distribution of anatomical connections, this suggests a link between feedforward signaling and gamma oscillations and feedback signaling and alpha/beta oscillations. A recent study provided direct evidence for these associations. The study characterized the information flow in human visual areas based on MEG data [48]. Specifically, measures of directed connectivity (such as **Granger causality**) indicated stronger connectivity in the gamma band from lower towards higher hierarchical areas (feedforward signal) whereas directed connectivity in the opposite direction (from higher to lower areas) is stronger in alpha/beta frequencies.

Taken together these findings indicate that neuronal oscillations and synchrony in distinct frequency bands serve the dynamic routing of information flow in the brain. Previously seemingly independent strands of research converged on the notion that alpha/beta oscillations mediate feedback signals whereas gamma oscillations mediate feedforward signals. In predictive coding frameworks of brain function, this might correspond to the signaling of predictions and prediction errors, respectively (Box 2). The involvement of neuronal oscillations in the flexible routing of information flow has been largely demonstrated and developed in the visual system. In the following section, we apply this concept to findings on neuronal oscillations and synchrony in the processing of pain.

## Neuronal Oscillations and the Experience of Pain

Most studies on the cerebral processing of pain have investigated responses to phasic pain stimuli in the range of milliseconds to seconds. These results are likely to apply to acute pain events that signal threat and promote protective behavior. Fewer studies have investigated the brain mechanisms of longer-lasting pain of months and years as a key feature of pathological chronic pain conditions. Furthermore, experimental studies on longer-lasting pain in the range of minutes (tonic pain) have investigated pain at timescales between those of phasic and chronic pain and are intended to represent an experimental approach towards chronic pain.

### Phasic Pain

EEG and MEG studies showed that brief noxious stimuli induce a complex spectral–temporal–spatial pattern of neuronal responses with at least three different components. First, pain stimuli evoke increased neural activity at frequencies below 10 Hz. These increases occur between 150 and 400 ms after stimulus application. They originate from the sensorimotor cortex and the frontoparietal operculum including the insula and secondary somatosensory cortex as well as from the mid-/anterior cingulate cortex. They correspond to the well-investigated pain-related evoked potentials 49, 50. Second, phasic pain stimuli transiently suppress oscillations at alpha and beta frequencies 23, 24, 51, 52. These suppressions are observed at latencies between about 300 and 1000 ms in the sensorimotor cortex and occipital areas 24, 51. Third, phasic pain stimuli induce oscillations at gamma frequencies over the sensorimotor cortex at latencies of between 150 and 350 ms 21, 22, 25.

The functional significance of the different components of pain-related brain activity is not yet fully understood. So far the evidence indicates that the components are differentially sensitive to different modulations of pain. Bottom-up modulations of pain by varying stimulus intensity (i.e., nociceptive information) influences all three components 21, 22, 25, 53, 54. Similarly, top-down modulations by varying attention affect all components 22, 51, 52, 54, 55. However, during spontaneous fluctuations of pain [21], pain modulations by music and music therapy [56], and repetitive painful stimulation [25] gamma oscillations are more closely related to pain intensity than the other components. By contrast, when pain is modulated by varying the expectation about the upcoming stimulus in the form of a placebo manipulation, evoked potentials and alpha suppressions are more closely related to pain than gamma oscillations [53]. Hence, bottom-up modulations affect all components of pain-related brain activity whereas different top-down modulations selectively modulate certain components. The available evidence does not yet allow more precise assignment of the different components to the manifold modulations of pain.

**Figure 1:**
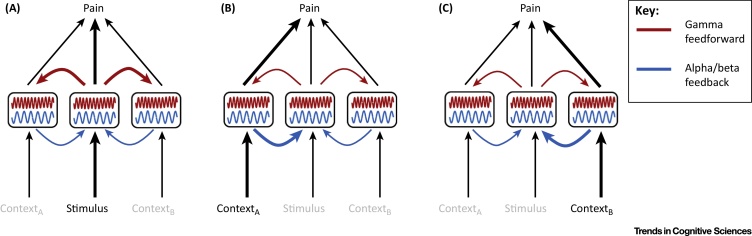
Key Figure: Flexible Routing of Information Flow in the Processing of Pain

The findings, however, indicate that brain activity at different frequencies provides different and complementary information about pain. Moreover, they indicate that there is no one-to-one correspondence between any frequency component of brain activity and pain, which extends the lack of specificity of brain activity for pain [15] to the frequency domain. Instead, the relationship between pain and brain activity is variable and context dependent. In the context of an involvement of oscillations in the flexible routing of information flow, the findings suggest that different contextual modulations of pain differentially change the information flow between the involved brain areas (Figure 1, Key Figure). For example, when pain is mostly driven by nociceptive processing, gamma oscillations in the somatosensory cortex may serve the feedforward signaling of sensory information to other brain areas involved in pain processing and behavioral responses to pain [57]. By contrast, when other processes such as affect or, evaluation dominate, the information flow is changed with gamma oscillations and feedforward signaling from the somatosensory cortex playing a rather minor role.

The assessment of brain responses to phasic painful stimuli shows the impact of contextual modulations on stimulus processing but not the mechanisms of the modulations. A straightforward approach to the disentangling of contextual processes from stimulus processing is the assessment of prestimulus activity, which cannot be contaminated by any stimulus-related processes. The few studies on this topic with respect to pain 58, 59, 60 suggest that ongoing oscillations play an important role in shaping pain perception. Specifically, the amplitude of prestimulus alpha oscillations over the sensorimotor cortex is negatively correlated with pain perception 59, 60. Correspondingly, attention to pain [52] and the expectation of analgesia [61] are associated with changes of alpha oscillations in the sensorimotor and prefrontal cortex, respectively. In addition, the amplitudes of prestimulus gamma oscillations are correlated with pain perception 58, 59, although the direction of the effect differed. Intriguingly, alpha and gamma oscillations together have a stronger predictive value than each component alone [59], which supports the view that they provide different and complementary information about feedforward and feedback signaling in pain processing.

Studies using **intracranial recordings** in a few patients with epilepsy investigated the significance not only of prestimulus oscillations but also of prestimulus connectivity between brain areas for pain. The results indicate that attention to pain changes the connectivity between pain-relevant brain areas at alpha and beta frequencies 62, 63, 64. Intriguingly, the analysis of directed functional (or effective) connectivity indicates that the information flow is flexibly changed by attention. Specifically, during attention to a painful stimulus the medial prefrontal cortex exerted causal influences on the primary sensorimotor cortex whereas during distraction the causal influences were reversed. These findings provide evidence for the context-dependent routing of information flow in the processing of pain (Figure 1). Moreover, the findings are well compatible with a role for synchrony at alpha and/or beta frequencies in the top-down signaling of contextual factors and/or, in a predictive coding framework (Box 2), predictions of pain. However, these promising findings originate from three patients and need replication and elaboration in further studies.

### Tonic Pain

The above-reviewed evidence relates to the processing of brief experimental pain stimuli. It is, however, unclear how these findings relate to the brain mechanisms of longer-lasting pain of months and years, which is the key feature of chronic pain. Experimental studies using longer-lasting tonic experimental pain stimuli in the range of minutes represent a step further in that direction. These studies have shown that tonic pain is associated with suppression of oscillations at alpha frequencies 65, 66, 67, 68, 69, 70, 71, 72, 73, 74, 75. However, as most mental processes suppress alpha oscillations, the specificity of this effect is unclear. Some studies have claimed it to be pain specific based on covariation of alpha oscillations and pain intensity 70, 71, 74. Another recent study showed that the suppression of alpha and beta oscillations during tonic pain is more closely related to stimulus intensity as a proxy for nociception than to the perceived pain intensity [73] indicating that these suppressions reflect stimulus processing rather than perception. In addition, several studies have recorded gamma oscillations during tonic pain 72, 73, 76. Intriguingly, during tonic pain gamma oscillations encoded pain rather than nociception [73]. Moreover, in contrast to phasic pain, they were not recorded over sensorimotor areas but over the medial prefrontal cortex [73].

Thus, during a few minutes of painful stimulation the encoding of pain shifts from gamma oscillations over brain areas encoding sensory processes to gamma oscillations over brain areas encoding emotional–motivational phenomena. These findings indicate that pain-related information flow might change not only with the behavioral context but also with the duration of pain. In the current framework of flexible routing of information flow (Figure 1), these findings suggest that during longer-lasting pain, signals from brain areas encoding emotional–motivational processes rather than from sensory brain areas dominate the processing and perception of pain. In a predictive coding framework (Box 2), this might indicate that longer-lasting pain does not generate prediction errors at the level of sensory processing but rather at the level of emotional–motivational processing.

### Chronic Pain

The analysis of oscillations and synchrony is conceptually promising and methodologically well suited for the investigation of ongoing processes such as chronic pain. However, remarkably few studies have addressed this topic and the results are not fully consistent (see [77] for a recent review). The most-noticed abnormality is an increase of theta oscillations in chronic pain patients (e.g., 34, 35). This phenomenon has been embedded in the framework of thalamocortical dysrhythmia 78, 79. This theory posits that abnormal thalamic theta oscillations play a crucial role in various neuropsychiatric disorders. In neuropathic pain deafferentation might cause these thalamic theta oscillations, which in turn entrain thalamocortical loops. At the cortical level, the abnormal theta oscillations are supposed to reduce lateral inhibition, which might result in abnormal gamma oscillations. Eventually, these abnormal gamma oscillations have been proposed to result in positive neurological and psychiatric symptoms including ongoing pain. The appeal of this framework is its internal coherence and there is some clinical and experimental evidence in favor of the concept 34, 35. However, other studies did not observe abnormal theta oscillations in chronic pain 80, 81. Moreover, as slowing of the peak alpha frequency in chronic pain 34, 35, 82, 83, 84, 85 has also been observed, abnormal amplitudes of theta oscillations might basically represent the unspecific slowing of EEG activity observed in many acute [86] and chronic [87] neuropsychiatric disorders.

A less-noticed finding is an increase of oscillations at alpha and beta frequencies 30, 34, 35, 85. This is in line with studies in animal models of chronic pain that showed broadband increases of oscillations from theta to beta frequencies in the primary somatosensory and medial prefrontal cortex 88, 89, 90. In particular, increases of beta oscillations were observed in frontal brain areas 34, 35, 85, 91, 92. Considering that beta oscillations are likely to serve feedback signaling 48, 93 and/or the signaling of predictions [94], this would be compatible with abnormal predictions playing a crucial role in chronic pain [95].

In summary, the data show mostly changes of theta and beta oscillations in chronic pain, the latter particularly in frontal brain areas. Considering disturbed integration of nociceptive and contextual processes in chronic pain, an abnormal balance of feedforward and feedback signaling and thereby an abnormal balance of oscillations at different frequencies might play an important role in chronic pain. However, the role of neuronal oscillations and synchrony in chronic pain is a largely unexplored field and the emerging concepts await further empirical testing.

## Concluding Remarks and Future Perspectives

Recent evidence has shown that oscillations and synchrony play a crucial role in the flexible routing of information flow in the brain. In particular, oscillations at gamma and alpha/beta frequencies have been shown to serve feedforward and feedback processing, respectively. The flexible routing of information flow might be particularly relevant in the processing of pain where the dynamic integration of sensory and contextual processes plays a crucial role (Figure 1). The results available so far are compatible with these concepts. It has been shown that there is no one-to-one correspondence between oscillations at any frequency or location and the subjective experience of pain, which extends evidence on the lack of specificity of pain-related brain activity [15] to the frequency domain. Instead, different modulations of pain are associated with distinct changes of neuronal oscillations indicating flexible, context-dependent routing of information flow. The available evidence does not so far allow more systematic mapping of the relationship between oscillations, cerebral information flow, and the experience of pain and its modulations. This lack of evidence is at least partly due to a lack of a systematic understanding of pain modulations [96]. Conversely, the systematic assessment of brain oscillations might represent a promising approach to establish a taxonomy [96] or ontology [97] of different types of pain modulation based on patterns of oscillations and cerebral information flow.

Chronic pain appears to be associated with abnormal oscillations at theta and beta frequencies. Although the specificity of these findings has remained unclear, at least part of them would be compatible with abnormal contextual feedback processes playing a central role in the pathology of chronic pain. More standardized approaches, larger patient samples, data-sharing initiatives, and more sophisticated and timely analysis strategies such as graph theory-based network analyses [98] are needed to further our understanding of the role of neuronal oscillations and flexible cerebral information flow in chronic pain. A better understanding of these processes might eventually help in the diagnosis and treatment of chronic pain. In particular, the assessment of oscillations and cerebral information flow might help to establish brain-based diagnostic markers of pain 99, 100. Moreover, the frequency-selective modulation of neuronal oscillations by brain stimulation techniques 101, 102 can determine causal influences between oscillations and behavior and might represent an option for the treatment of pain.

Furthermore, considering the preeminent role of the integration of contextual and sensory information in the processing of pain, an application of predictive coding frameworks (Box 2) to the processing of pain is obvious. In such a framework, contextual and nociceptive information might be conceptualized as predictions and sensory evidence, respectively. Pain thereby results from the comparison and adjustment of predictions, sensory evidence, and prediction errors rather than directly from nociceptive information. Accordingly, it has recently been proposed that predictive coding represents a suitable and testable model of pain processing 7, 103, 104, 105. Paradigmatically, a predictive coding model for pain and placebo analgesia has been presented [103]. In this model placebo-induced treatment expectations were conceptualized as feedback-mediated predictions, which modulate pain by changing the balance of feedback and feedforward processes at different levels of a neural processing hierarchy. It will be intriguing to extend this model to other modulations of pain. Moreover, considering the relationship of predictions and prediction errors with alpha/beta and gamma oscillations, respectively 44, 94, 106, 107, the assessment of oscillations could provide novel insights into predictive coding processes related to pain. This is even more appealing as abnormally precise predictions [95] and/or abnormal updating of predictions [7] might play an important role in the pathology of chronic pain.

Thus, based on recent progress in our understanding of neuronal oscillations, their systematic assessment might provide a unique window onto the dynamics of cerebral information flow and related predictive coding processes underlying the experience of pain in health and disease.

Outstanding Questions Recent studies discuss the significance of interactions of oscillations at different frequencies (i.e., cross-frequency coupling). What is the role of cross-frequency coupling in pain? In particular, how do infraslow fluctuations observed by fMRI relate to oscillations at higher frequencies in the processing of pain?

Pain modulations can be harnessed for pain therapy. However, a systematic understanding of pain modulations is so far lacking. Can the assessment of oscillations and patterns of cerebral information flow help to establish a brain-based taxonomy of pain modulations?

It is tempting to relate the interaction between sensory input, contextual information, and pain to predictive coding frameworks of brain function. How can this relationship be specified and experimentally tested? What are the consequences for the understanding of pain and chronic pain?

The analysis of oscillations is conceptually and methodologically well suited for the investigation of the brain mechanisms of chronic pain. However, evidence on the role of oscillations and synchrony in chronic pain is remarkably limited. Can timely network analyses of EEG, MEG, and fMRI data specify abnormalities of oscillations and synchrony underlying chronic pain?

Subcortical areas including the ventral striatum, amygdala, and hippocampus play an important role in chronic pain. Although neuronal oscillations from these areas are well known they have not so far been investigated during pain. How can subcortical oscillations be recorded and how do they integrate in patterns of cerebral information flow?

Recent studies discuss the use of patterns of brain activity as markers of pain. Can patterns of neuronal oscillations and synchrony serve as diagnostic and/or prognostic markers of pain? Can we target neuronal oscillations and synchrony for pain therapy using pharmacological, behavioral, neuromodulatory, or neurofeedback approaches?

## Box 1 Infraslow Fluctuations and Pain

In resting-state fMRI data, infraslow fluctuations of blood oxygenation level-dependent (BOLD) signals below 0.1 Hz have been observed [108]. The synchronization of these infraslow fluctuations across brain areas defines **intrinsic brain networks** [108]. Accumulating evidence indicates that particularly the activities of the **default mode, salience, and sensorimotor networks** are changed in chronic pain 26, 27, 28, 29. The findings show changes of connectivity not only within but also across [109] these networks in chronic pain. Moreover, changes of the frequency pattern 26, 27 and the variability [110] of infraslow fluctuations have been observed. Thus, the analysis of infraslow fluctuations is a promising approach to advance our understanding of the brain mechanisms of chronic pain. However, changes of infraslow fluctuations of BOLD signals are found not only in pain but in many neuropsychiatric disorders [111] so that the disease and symptom specificity of the observed changes remains an important question.

Two physiological aspects of intrinsic brain networks might be of particular interest in the processing of pain. First, intrinsic brain networks relate to slowly propagating waves of neuronal activity at a timescale of about 1 s for the whole cortex 112, 113. These waves provide subthreshold depolarization to individual neurons indicating modulation of neuronal excitability [114]. Changes of infraslow BOLD fluctuations in chronic pain might therefore indicate impairment of slowly propagating waves. As slowly propagating waves are intimately linked to **slow waves** during sleep and memory consolidation [115], changes of infraslow BOLD fluctuations might relate to abnormal learning and memory consolidation processes in chronic pain [13]. Second, infraslow fluctuations are linked to fluctuations of faster oscillations at alpha and gamma frequencies 116, 117 indicating cross-frequency coupling with infraslow fluctuations shaping faster oscillations. In chronic pain, abnormal infraslow fluctuations, particularly in the salience and default mode networks 26, 27, 28, 29, might interfere with faster oscillations and the flexible routing of information flow in the brain.

In summary, infraslow fluctuations provide a mechanistic framework that connects slow wave propagation and learning processes to oscillations at higher frequencies and flexible cerebral information flow. Abnormalities of infraslow fluctuations and their synchronization might signal abnormal slow wave propagation and related learning processes and contribute to abnormal routing of information flow in chronic pain.

## Box 2 Predictive Coding

Predictive coding is a framework of brain function that states that the brain is not a passive recipient of information but generates and optimizes predictions about the environment 118, 119. These predictions are continuously compared against sensory evidence and discrepancies produce prediction errors that serve to optimize future predictions. In this way the brain efficiently allocates resources to events that are behaviorally relevant and useful for updating predictions (i.e., learning processes). These processes are implemented in a hierarchical processing model in which predictions and prediction errors are passed in feedback and feedforward directions, respectively. At every level of the hierarchy, prediction errors are minimized by optimizing predictions. In this optimization process, the influences of predictions and sensory evidence are weighted according to their precision.

In the cortex predictive coding processes are likely to be implemented by various neuronal populations [44]. In particular, predictions have been related to infragranular neurons and feedback connections while prediction errors have been related to supragranular neurons and feedforward connections [44]. In light of the predominance of alpha/beta and gamma oscillations in infra- and supragranular layers, respectively, the relation of predictions and prediction errors to neuronal oscillations at alpha/beta and gamma frequencies, respectively, is obvious 44, 94. Recent studies have provided the first experimental evidence for such relationships 106, 107.

## Glossary

Default mode, salience, and sensorimotor networks: important intrinsic brain networks that are particularly active during rest and during the detection of and orientation to salient events and sensorimotor processes, respectively. These networks have been shown to be involved in the processing of pain.
Granger causality: a measure of the causal relationship between two time series. In EEG, MEG, and intracranial recordings, it is often taken as a measure of the causal relationship between neural signals originating from different locations in the brain.
Infra- and supragranular layers: the largest part of the cerebral cortex, termed the isocortex, is characterized by a six-layered structure. The different layers contain characteristic distributions of neuronal cell types and connections. From outside to inside, the layers are numbered with Roman numerals from I to VI. Layer IV is termed the internal granular layer. Layers I–III are summarized as the supragranular layers and V and VI as the infragranular layers. The supra- and infragranular layers differ in their patterns of feedforward and feedback connections.
Intracranial recordings: neurophysiological recordings of brain activity obtained either from electrodes placed on the cortex (electrocorticography) or from electrodes inserted in the brain (LFPs). In humans intracranial recordings can be obtained during surgery or after surgical implantation of electrodes.
Intrinsic brain networks: sets of brain areas that exhibit synchronous activity in fMRI recordings during the resting state.
Nociception: the neural process of encoding noxious stimuli.
Nociceptive: related to noxious stimuli.
Slow waves: slow oscillations at frequencies below 3 Hz observed in EEG and LFP recordings. Slow waves are mostly observed during sleep and are likely to play an important role for memory consolidation.
